# A Numerical Investigation on the Collision Behavior of Polymer Droplets

**DOI:** 10.3390/polym12020263

**Published:** 2020-01-24

**Authors:** Lijuan Qian, Hongchuan Cong, Chenlin Zhu

**Affiliations:** College of Mechanical and Electrical Engineering, China Jiliang University, Hangzhou 310018, China; P1701085211@cjlu.edu.cn (H.C.); zhuclgary@foxmail.com (C.Z.)

**Keywords:** polymer liquid, the surface tension, the coupled level-set and volume of fluid method

## Abstract

Binary droplet collisions are a key mechanism in powder coatings production, as well as in spray combustion, ink-jet printing, and other spray processes. The collision behavior of the droplets using Newtonian and polymer liquids is studied numerically by the coupled level-set and volume of fluid (CLSVOF) method and adaptive mesh refinement (AMR). The deformation process, the internal flow fields, and the energy evolution of the droplets are discussed in detail. For binary polymer droplet collisions, compared with the Newtonian liquid, the maximum deformation is promoted. Due to the increased viscous dissipation, the colliding droplets coalesce more slowly. The stagnant flow region in the velocity field increases and the flow re-direction phenomenon is suppressed, so the polymer droplets coalesce permanently. As the surface tension of the polymer droplets decreases, the kinetic and the dissipated energy increases. The maximum deformation is promoted, and the coalescence speed of the droplets slows down. During the collision process, the dominant pressure inside the polymer droplets varies from positive pressure to negative pressure and then to positive pressure. At low surface tension, due to the non-synchronization in the movement of the interface front, the pressure is not smooth and distributes asymmetrically near the center of the droplets.

## 1. Introduction

The collision dynamics of droplets in a gas environment are of great importance in the industrial processes related to spraying, such as spontaneous combustion gel propellants in missile propulsion systems [1], 3D inkjet printing in textile printing [2,3,4], and polymer–solvent sprays in chemical pharmaceutical engineering [5]. In particular, in order to develop better-performing products, the surfactants are often used to change the surface tension of the fluid without changing other properties. During dairy processing, such as the preparation of emulsions in the field of cosmetics, the surfactants are used to accelerate the coalescence process [6]. During the ASP flooding process, the surfactants are added to improve the oil recovery efficiency [7]. In the research of Fortelný et al. [8], theories of the flow-induced coalescence providing equations for collision efficiency were discussed, and approximate analytic expressions reliably describing the dependence of the collision efficiency on system parameters were presented. Available theories describing the competition between the droplet breakup and coalescence in flow were summarized and approximations used in their derivation were discussed. The review addressed problems with the applicability of available theories on the prediction of the droplet size evolution during mixing and processing of immiscible polymer blends, which have not been broadly discussed so far.

For binary Newtonian droplet collisions, the current studies [9,10,11,12,13,14,15,16,17,18,19,20,21,22,23] are mainly focused on two aspects: from binary low-viscosity droplet collisions to binary high-viscosity droplet collisions, and from binary homogeneous-droplet collisions to binary heterogeneous-droplet collisions. Jiang et al. [9] experimentally studied the water and n-alkane droplet collision process. Results showed that the energy dissipation in the radial deformation phase was independent of the viscosity coefficient, but depended on the viscosity coefficient in the phase of the small-amplitude free oscillation of the droplets. Willis and Orme [10] studied binary viscous droplet collisions experimentally in a vacuum environment. It was considered that the percentage of dissipated energy until the instant of maximum deformation increased with the increasing fluid viscosity when the Oh number was greater than 0.03 Pa·s. This observation has been verified by the research of Gotaas et al. [11] and Dai et al. [12], but it contradicts the previous results by Jiang et al. [9]. Kuschel and Sommerfeld [13,14] further experimentally studied the collision process of the high viscous solution droplets. The boundary models were derived and revealed that the dependence of the critical Weber number on the dynamic viscosity. By changing the surface tension of the droplets, Chen et al. [15] experimentally studied the collision outcomes of binary immiscible droplets. An “overlaying” action for the drop with the smaller surface tension to go around the surface of the drop with the larger surface tension occurred in the near-head-on collision process, which made the reflexive separation more difficult to occur. Planchette et al. [16] experimentally investigated the onset of fragmentation after binary immiscible droplet collisions. Three main phenomena—full encapsulation, head-on fragmentation, and off-center fragmentation—were observed after the collisions. Based on the capillary instability and an energy balance, a scaling law for the evolution of the threshold impact velocity was established for head-on collisions. The research of Pan et al. [17] identified the key mechanisms governing the impact dynamics of surfactant-coated droplets in the air and imply the potential of using a small amount of surfactant to manipulate impact outcomes. Compared to the Newtonian liquids, relatively few studies [24,25,26,27,28,29,30] have been conducted for the non-Newtonian liquids, which are of relevance to, for example, polymers. For the non-Newtonian liquid, the effects of the viscosity on the deformation process and the boundary model of the droplets are investigated. Finotello et al. [24] experimentally studied the effect of viscosity on the collision outcomes of binary droplets by the Herschel–Bulkley (H–B) model. They found that, in the collision process, the kinetic energy was dissipated due to viscous flow, and thus the critical Weber number for the reflexive separation increased with the Ohnesorge number. By combining the experimental results [24], Finotello et al. [25] further experimentally investigated the collision behaviors of binary shear-thinning droplets and presented a complete regime map for collision boundaries based on the Weber number (We) and impact parameter (B).

In the experiments, for the formation of high-quality and stable droplets, the transient visualization of the collision droplets is difficult to realize, so numerical simulation work is needed to compensate for this technical deficiency. Amani et al. [19] simulated head-on and off-center binary droplet collisions in all the regimes [18] using a conservative level-set method. A novel lamella stabilization approach was introduced to numerically resolve the thin lamella film. The in-depth energy analysis was performed for each case covering a wide range of collision regimes, which provides more insight into the collision process. Jiang et al. [26] simulated the collision process between two miscible/immiscible micro-droplets by a modified smoothed particle hydrodynamics (C-SPH) method. Results showed that the collision deformation process of polymer droplets was much more complicated than that of the Newtonian droplets. For the non-Newtonian liquid, based on the volume of fluid (VOF) method, Motzigemba et al. [27] conducted the numerical simulation of binary droplet collisions using Newtonian and non-Newtonian fluid. They found that the deformation of the colliding droplets was larger for the shear-thinning fluid compared to the Newtonian fluid. The dimensionless time until the collision complex attained its maximum expansion and was independent of the viscosity but related to the Weber number. And the simulated velocity fields showed that at regarding all the collision processes, elongational flow dominated. Based on the investigation of Motzigemba et al. [27], the simulation results of Focke and Bothe [28] showed that the collision dynamics of the shear-thinning fluid could be reproduced by that of the Newtonian fluid with an effective viscosity because only during an initial phase of the collision the viscous forces were important. Liu et al. [29] simulated binary head-on collision of gel propellant droplets by the VOF method. Phenomena of rebound, coalescence, and reflexive separation of droplets were investigated. Results showed that the rebound of droplets was determined by the Weber number and the viscosity of the fluid. The minimum central thickness of the droplets appeared later than its maximum deformation in the low Weber number, which was on the contrary to that in the high Weber number. Moreover, the complicated flow fields showed that the maximum shear rate appeared at the point where the flow was redirected and accelerated. Sun et al. [30] simulated binary non-Newtonian droplet collisions in a two-dimensional coordinate system by the lattice Boltzmann (LB) method. The Carreau–Yasuda (CY) model was employed to describe the effect of the non-Newtonian rheology on the coalescence, separation, and internal mixing of binary droplets. Results showed that, with the increasing non-Newtonian effects, the internal mixing, deformation, and separation of the colliding droplets was promoted for the shear-thinning fluid, and the permanent coalescence was promoted for the shear-thickening fluid because the increased viscous loss suppressed the droplet separation. In addition, for binary droplets with a larger shear-thinning disparity, the large-scale internal mixing was promoted, while the collision between a shear-thinning droplet and a shear-thickening droplet was found to facilitate the coalescence and mixing.

In view of the above research, compared to the Newtonian liquid, the research on binary non-Newtonian droplet collisions is mainly focused on the effect of the viscosity on the deformation process and the boundary model of the droplets. The investigation of the influence of the surface tension on the collision behavior of the non-Newtonian droplets and the detailed discussion of the internal flow fields and the energy evolution of the droplets are still lacking. Therefore, in this paper, based on the coupled level-set and volume of fluid (CLSVOF) method and adaptive mesh refinement (AMR), the effects of viscosity and surface tension on the collision behaviors of the non-Newtonian droplets will be investigated.

The structure of the paper is the following. Section 2 gives the numerical framework, and the CLSVOF method and power-law viscosity model are also introduced. Section 3 is the verification, including the computational setup, verification of the grid independence, and experimental verification; the numerical simulation is quantitatively compared with the experimental results of Qian et al. [18]. Section 4 is the results and discussion; the collision process of the polymer droplets is compared with that of the Newtonian droplets. Then the effects of the surface tension on the collision results of the polymer droplets are studied. Finally, the internal flow field (velocity and pressure) and the energy evolution of the droplets are discussed in detail. In Section 5 the conclusions are summarized.

## 2. Numerical Framework

### 2.1. Governing Equations

The coupled level-set and volume of fluid (CLSVOF) method is used in our paper, which can accurately capture the complex process of the interface evolution and has good mass conservation [31,32,33]. Two-phase flows are supposed to be isothermal, immiscible, and incompressible. The governing equations, including the continuity, momentum, and phase-fraction equations, can be written as

(1) ∇·U=0

(2) $$\frac{\partial \rho U}{\partial t}+\nabla \cdot \left(\rho UU\right)=-\nabla p+\nabla \cdot \tau +F_{\sigma }$$

(3) $$\frac{\partial \alpha }{\partial t}+\nabla \cdot \left(\alpha U\right)=0$$

where ρ is the mixture density, U is the velocity vector of the flow field, p is the pressure, τ is the stress tensor defined as $\tau =\mu \left(\nabla U+\nabla U^{T}\right)$, in which μ is the mixture droplet dynamic viscosity. $F_{\sigma }$ is the volume force. The mixture density ρ and viscosity μ adopting phase fraction α as a weight coefficient can be calculated as the transition of two-phase physical properties in the interface region:

(4) $$\rho =\alpha \rho_{l}+\left(1-\alpha \right)\rho_{g}$$

(5) $$\mu =\alpha \mu_{l}+\left(1-\alpha \right)\mu_{g}$$

In the original VOF function (3), the additional compression term $\nabla \cdot \left(U_{c}\alpha \left(1-\alpha \right)\right)$ is introduced to sharpen the interface [34]. The improved VOF equation can be written as

(6) $$\frac{\partial \alpha }{\partial t}+\nabla \cdot \left(\alpha U\right)+\nabla \cdot \left[U_{c}\alpha \left(1-\alpha \right)\right]=0$$

where $U_{c}$ is the compression velocity to suppress diffusion of the interface, which is defined as

(7) $$U_{c}=min\left(c_{\alpha }\left|U\right|,max\left(\left|U\right|\right)\right)\cdot \frac{\nabla \alpha }{\left|\nabla \alpha \right|}$$

where $c_{\alpha }$ is the compression coefficient, which determines the compression magnitude. It is generally greater or equal to 1. The term $\frac{\nabla \alpha }{\left|\nabla \alpha \right|}$ is introduced to present the convection of volume fraction function normal to the interface. Then the term α(1−α) can be used to ensure itself invalid in the outside of the interfacial area and the divergence operating guarantees mass conservation in terms of the entire compression term. The phase-fraction transport equation of the VOF function α is solved in the whole computational domain as a guarantee of the mass conservative nature.

Furthermore, the level-set (LS) function ψ is introduced to ensure the interface smoothness by calculating smoother curvature. The method initializes the LS field by using $\psi_{0}=\left(2\alpha -1\right)\Gamma$ as the prime guess value, where Γ is a small non-dimensional number whose value depends on the minimum mesh size Δx. The LS function $\psi_{0}$ is a signed function, which can then be re-distanced by the reinitialized equation,

(8) $$\frac{\partial \psi }{\partial \tau }=S\left(\psi_{0}\right)\left(1-\left|\nabla \psi \right|\right)$$

where *τ* is the artificial time step and $S\left(\psi_{0}\right)$ is a sign function defined with $S\left(\psi_{0}\right)=\frac{\psi_{0}}{\left|\psi_{0}\right|}$. The solution of ψ converges to |∇ψ|=1, which is a signed distance function around the interface, and the interface position is defined at the contour-line ψ=0. The number of iterations ($\psi_{corr}$) meets the following term:

(9) $$\psi_{corr}=\frac{\varepsilon }{\Delta \tau }$$

where *ε* is the interface thickness. The LS function can enable the accurate calculation of the interface normal by $n=\frac{\nabla \psi }{\left|\nabla \psi \right|}$. The smoother curvature is derived by κ=∇·n. Therefore, the volumetric surface tension force can be calculated:

(10) $$F_{\sigma }=\sigma \kappa \left(\psi \right)\delta \left(\psi \right)\nabla \psi$$

where *σ* is the surface tension coefficient and *δ* is the Dirac function used to confine the influence of the surface tension to a narrow area around the interface.

(11) $$\delta \left(\psi \right)=\{\begin{array}{cc} 0, & \left|\psi \right|>\varepsilon \\ \frac{1}{2\varepsilon }\left(1+cos\left(\frac{\pi \psi }{\varepsilon }\right)\right), & \left|\psi \right|\leq \varepsilon \end{array}$$

### 2.2. Power-Law Viscosity Model

In this paper, we will choose non-Newtonian water/carboxymenthylcellulose (CMC) solutions, because it has strong shear-thinning behavior and low elastic effects. The power-law model is presented as follows:

(12) $$\mu =K{\dot{\gamma }}^{n-1}$$

with *μ* the apparent viscosity, $\dot{\gamma }$ the shear rate, *K* the consistency index (*K* = 6.69), and *n* the power-law index of the fluid (*n* = 0.2).

In the polymeric system, at low or high shear rates, the apparent viscosity approaches a Newtonian plateau, where the viscosity is dependent on shear rate. The dynamic viscosity at zero and infinite shear rates can be defined as

(13) $$\mu_{0}={lim}_{\dot{\gamma }\to 0}\mu$$

(14) $$\mu_{\infty }={lim}_{\dot{\gamma }\to \infty }\mu$$

For the shear-thinning liquid, the apparent viscosity decreases with the increasing shear-rate. Through Equation (12), the effective viscosity at the characteristic shear rate $\dot{\gamma }=V_{r}/D$ can be calculated as

(15) $$\mu =K{\left(V_{r}/D\right)}^{n-1}$$

## 3. Verification

### 3.1. Computational Setup

**Figure 1:**
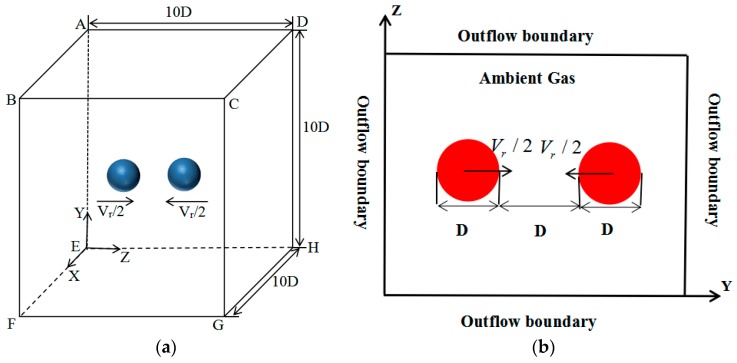
Schematics of the computational domain and the initial state of the binary droplets. (**a**) Schematic of the computational domain; (**b**) schematic of binary droplet collisions projected to the Y–Z plane.

As shown in Figure 1, two spherical droplets generated by the adaptive mesh refinement (AMR) are initially located in the three-dimensional coordinate system. The computational domain (*x* × *y* × *z*) is 10*D* × 10*D* × 10*D* and the grid number (*x* × *y* × *z*) is 80 × 80 × 80. The initial diameter (*D*) is 336 μm. Pressure-outlet boundary conditions are applied in all the computational domain planes.

The five dimensionless numbers for the collision dynamics of binary droplets in this work were the Weber number (*We*), the Reynolds number (*Re*), the Ohnesorge number (*Oh*), the Capillary number (*Ca*), and the dimensionless time ($t^{*}$), respectively defined as: $We=\rho_{l}V_{r}^{2}D/\sigma$, $Re=\rho_{l}V_{r}D/\mu$, $Oh=\mu /\sqrt{\rho_{l}D\sigma }$, $Ca=\mu V_{r}/\sigma$, $t^{*}=\left(t-t_{0}\right)V_{r}/D$, where $\rho_{l}$ is the liquid density, σ is the surface tension coefficient, $V_{r}$ is the relative velocity vector of the colliding droplets, and *t*_0_ is the initial collision time. For non-Newtonian liquid, the liquid viscosity *μ* is the effective viscosity, as shown in Equation (12). The Ohnesorge number at the effective viscosity is defined as the effective Ohnesorge number (Oh_eff_).

Based on the experimental investigation of Qian et al. [18], the density of tetradecane liquid $\rho_{l}$ is 758 kg/m^3^, the surface tension *σ* is 0.026 kg/s^2^, and the viscosity *μ* is 2.128 × 10^−3^ Pa·s. The experimental case at *We* = 61.4 and *Oh* = 0.0262 is conducted to compare with the simulation results of the non-Newtonian liquid. 

### 3.2. Verification of Grid Independence

**Figure 2:**
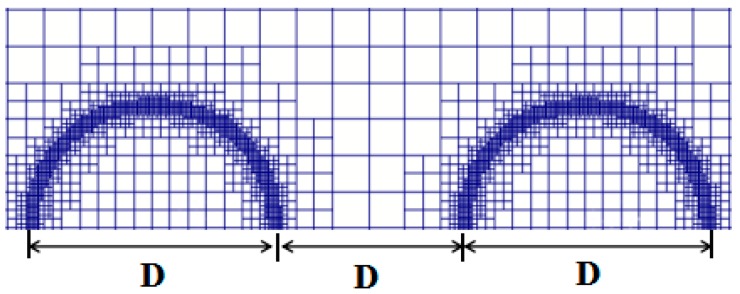
Initial droplets and adaptive mesh.

In the present study, the adaptive mesh refinement (AMR) is used, as shown in Figure 2. The numerical resolution related to the cell refinement level of the droplet is *D*/Δ*x*. The minimum cell size Δ*x* is defined as $L_{D}/2^{L_{\max}}$, where *L*_D_ is the side length of a square, and *L*_max_ is the cell refinement level. 

**Figure 3:**
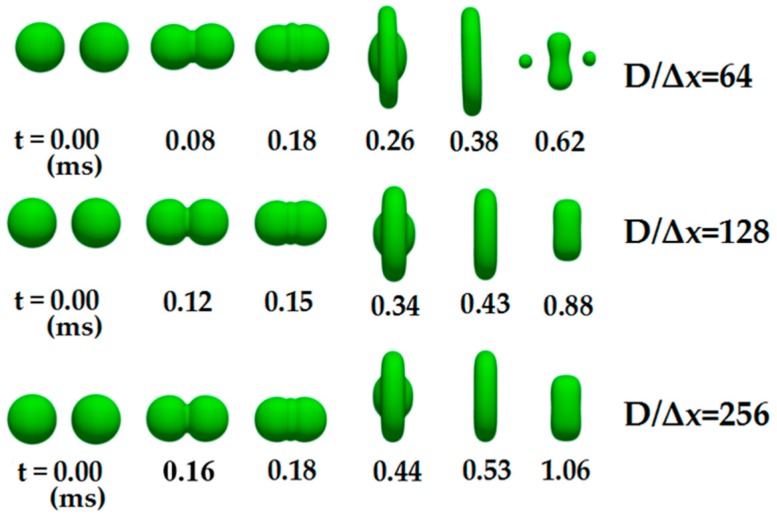
Verification of the grid independence under three different cell refinement levels.

The contour change diagrams of binary droplets related to three numerical resolutions are given in Figure 3. As can be seen, the higher the cell refinement level, the more accurate the numerical results. When the numerical resolution is *D*/Δ*x* = 64, the droplet is broken in the stage of the axial extension due to the instability of the interface. When the numerical resolution *D*/Δ*x* = 128 and *D*/Δ*x* = 256, our numerical results have a slight difference, but the time consumption in computation increases several times. Therefore, the intermediate value *D*/Δ*x* = 128 is adopted in all of the following simulations.

### 3.3. Experimental Verification

**Figure 4:**
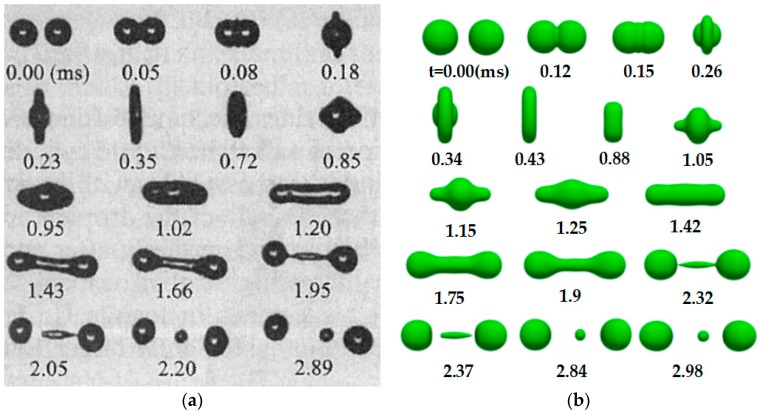
Comparison with the experimental images of Qian et al. [18] and our simulation result under the condition of *We* = 61.4 and *Oh* = 0.0262. (**a**) Experiments. (**b**) Simulation results.

Figure 4 shows the simulation results compared with the experimental case of Qian et al. [18] under the condition of *We* = 61.4 and *Oh* = 0.0262. The shape evolution of binary droplet collisions is in good agreement with the experimental results.

## 4. Results and Discussion

For the non-Newtonian liquid (CMC), the liquid density $\rho_{l}$ is 1000 kg/m^3^, and the surface tension *σ* is 0.07 kg/s^2^ [27]. The Ohnesorge number (Oh) corresponding to the viscosity at the zero shear-rate is 0.524, which is 20 times larger than that of Newtonian liquid. Table 1 gives the computational parameter settings of the simulation cases.

### 4.1. A Comparison of the Collision Behavior of the Droplets of the Newtonian and Shear-Thinning Liquid

**Figure 5:**
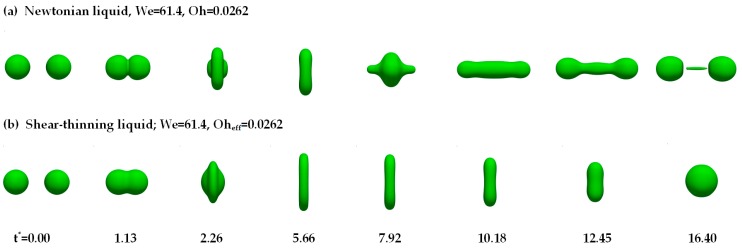
Comparison of binary droplet collisions of Newtonian and shear-thinning liquid.

Figure 5 shows the collision process of the droplets using the Newtonian liquid and the shear-thinning liquid.

In Figure 5, the effective Ohnesorge number (*Oh*_eff_) of the shear-thinning liquid is the same as that of the Newtonian liquid. For the Newtonian liquid, binary droplets coalesce and form an obvious liquid bridge at t* = 1.13. During the radial extension process (t* < 5.56), a thin dimpled disk with a small radius of curvature at its edge is formed. Subsequently, due to the surface tension, the disk contracts radially. From t* = 7.92 to 12.45, the droplets extend axially into the cylinder, and two ends of the cylinder gradually spheroidize gradually with the contraction of the connecting neck. At t* = 16.40, through the action of the end-pinching mechanism, the two boundary droplets separate and the ligament structure appears in the middle [18]. For the shear-thinning liquid in Figure 5b, the mechanism of ligament breakup does not occur and the permanent coalescence is promoted. During the radial extension process (t* < 5.56), the polymer droplets attain a larger radial maximum diameter than the Newtonian droplets, which also can be found in the experiments of Motzigemba et al. [27]. However, due to the stronger surface tension effect, the polymer droplets eventually shrink into a sphere, and the axial extension is suppressed due to the increased viscous resistance, resulting in no separation after the droplet collision.

**Figure 6:**
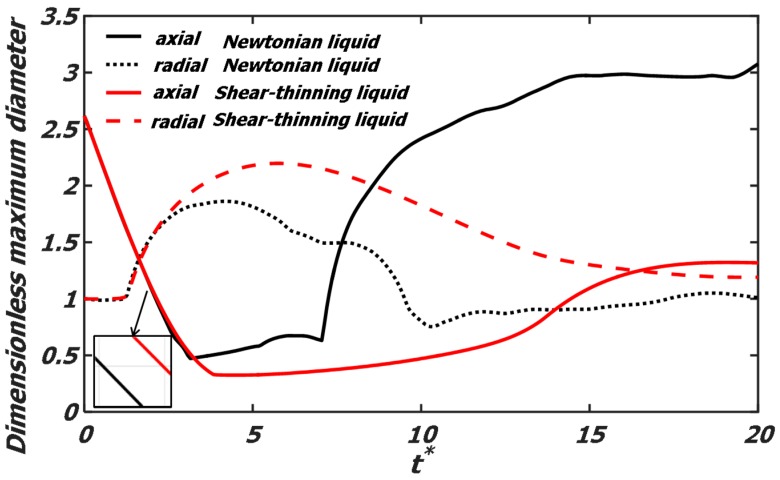
Dimensionless maximum diameter evolution of the droplets. The Newtonian droplets at *We* = 61.4, *Oh* = 0.0262; and the shear-thinning droplets at *We* = 61.4, *Oh*_eff_ = 0.0262. The solid line is the dimensionless maximum axial diameter, and the dotted line is the dimensionless maximum radial diameter.

Figure 6 gives the variation of the maximum axial and radial diameter of the droplets, which is dimensionalized by the initial diameter (*D*) of the droplets. At the initial stage, the Newtonian droplets coalesce faster than the shear-thinning droplets. For binary polymer droplets collision, the maximum deformation of the droplets is promoted. At the axial extension stage, due to the sufficient kinetic energy, the dimensionless axial diameter of the Newtonian droplets increases rapidly, so the droplets recover the spherical shape faster. When the dimensionless axial diameter continues to increase to 3, the reflexive separation occurs. While for the shear-thinning liquid, due to the increased viscous force, the time for the droplets to return to the sphere is delayed.

### 4.2. The Surface Tension Effect

**Figure 7:**
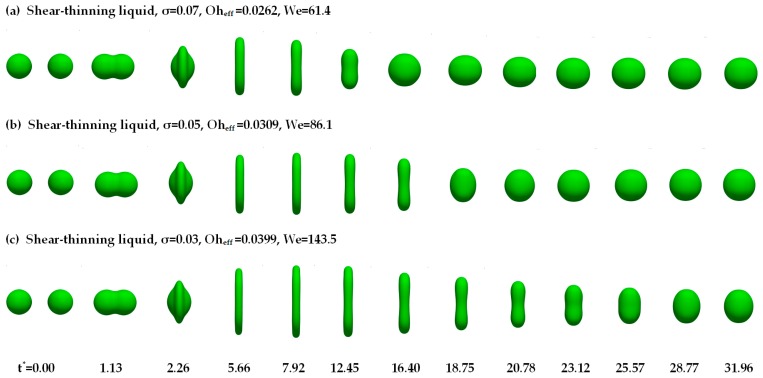
The morphological evolution of colliding droplets with a different surface tension.

In Section 4.1, no separation occurs during the collision process of the polymer droplets. In this section, comparisons of the collision behavior of the droplets under different surface tension conditions are given in Figure 7.

In Figure 7, after the calculation over a long time span, for the final outcome of droplet collision, the permanent coalescence occurs. It is obvious that the deformation processes in the early stage (t* < 5.66) are similar. The two droplets collide with each other, form a Saturn shape, and the droplets extend rapidly in the radial direction subsequently. The visible difference can be observed that the larger the surface tension is, the faster the merging droplets reach the maximum radial diameter. Due to the surface tension, the disk-like droplets then are radially contracted to a spherical droplet. The larger the surface tension is, the faster the radial contraction of the droplets is. At large surface tension (*σ* = 0.07), the disk-like droplets turn into the oblate spheroid shape quickly. At moderate surface tension (*σ* = 0.05), the droplets turn into a sphere smoothly, while at low surface tension (*σ* = 0.03), the droplets are an ellipsoid shape.

**Figure 8:**
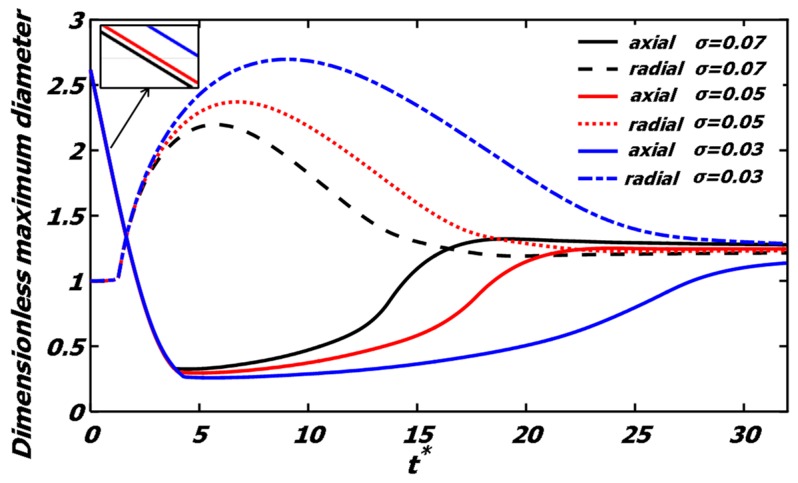
Dimensionless maximum diameter evolution of the polymer droplets under different surface tension conditions (*σ* = 0.07, *We* = 61.4, *Oh*_eff_ = 0.0262; *σ* = 0.05, *We* = 86.1, *Oh*_eff_ = 0.0309; *σ* = 0.03, *We* = 143.5, *Oh*_eff_ = 0.0399). The solid line is the dimensionless maximum axial diameter, and the dotted line is the dimensionless maximum radial diameter.

Figure 8 gives the time evolution of the dimensionless maximum diameter of the droplets under different surface tension. At the initial stage, the coalescence of the colliding droplets can be promoted by the larger surface tension. As the surface tension decreases, the droplets reach a larger maximum deformation, but the time for the drops to reach the maximum deformation and recover the spherical shape is delayed. The research of Motzigemba et al. [27] showed that the time for maximum expansion of the collision complex had a strictly monotonic dependency on the Weber number.

### 4.3. The Internal Flow Fields

**Figure 9:**
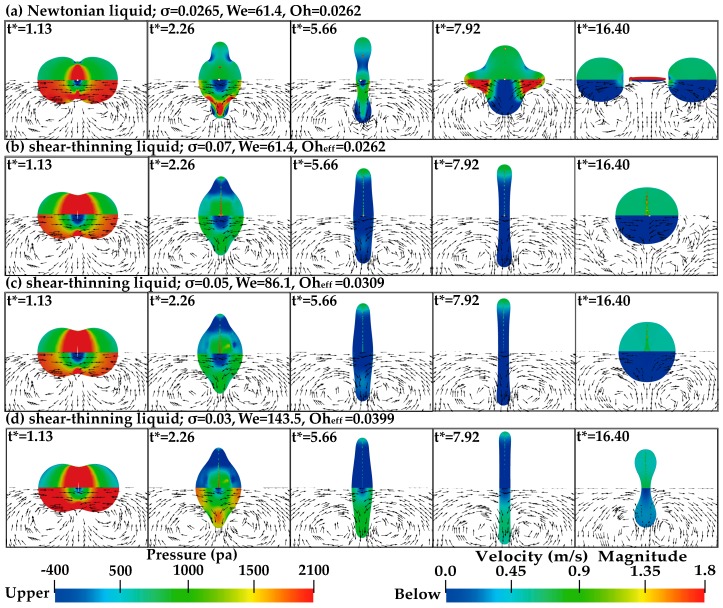
Evolution of the flow fields (pressure, velocity) for the *Y*–*Z* plane at *X* = 0.00168 for the droplets of the Newtonian and shear-thinning liquid. The pressure field is at the top and the velocity field is at the bottom of each graph.

Figure 9 gives the pressure field and the velocity field of the droplets of Newtonian and shear-thinning liquid. In the velocity field of the droplets, the equal length velocity vectors are used to describe the formation and flow direction of the vortex rings, and the velocity contour is used to illustrate the magnitude of the velocity.

For all the cases in Figure 9, as two droplets approach each other, due to the forward movement of the interface front, the gas is squeezed out to form a sheet jet and surrounding gas is entrained into this jet, forming vortex rings on both sides of the jet. At the collision moment of the droplets, the high-pressure region appears near the center of the droplets, and a stagnant flow region is formed. In this flow region, the pressure is greater than the surface tension, and the droplets begin to extend radially [9]. In Figure 9a, for the Newtonian liquid, the droplets coalesce immediately after contact at t* = 1.13. At the radial extension stage (from t* = 1.13 to 5.66), the droplets extend radially at a higher speed. The negative pressure is mainly distributed in the concave area of the neck of the droplets. After the droplets reach the maximum deformation, the droplets begin to contract due to the surface tension, and the negative pressure region increases. At this point, the radial flow is forced to start flowing inward, while the flow is still outward, so the stagnant flow region can be observed near the bulbous ends of the droplets, as shown at t* = 5.66. Subsequently, due to the surface curvature, the vortex to change its rotational direction, the droplets extend axially with a higher speed at the end of the bell, and the pressure on the concave area of the droplets decreases, as shown at t* = 7.92. At t* = 16.40, because the internal flow has enough kinetic energy to overcome the surface tension force, the fluid continuously converges at both ends of the droplets, gradually forming the spherical structure, and a liquid ligament appears between the two separated droplets. The maximum pressure can be observed at the ligament, and the maximum velocity can be observed at the pinch-off point. For the shear-thinning liquid in Figure 9b, at the initial collision moment (t* = 1.13), the droplets do not coalesce completely. A narrow air gap channel is formed in the center of the collision plane, and then the air gap is divided into many small segments, which are involved in the coalesced droplets. Compared with the Newtonian liquid in Figure 9a, due to the large deformations of the polymer droplets, a broader high-pressure region forms near the center of the droplets at t* = 1.13. During the collision process (from t* = 2.26 to 7.92), the distribution of the positive and negative pressure region of the droplets is also different from that of the Newtonian droplets; that is, the negative pressure region distributes inside of the polymer droplets, while the positive pressure region distributes at both ends of the droplets. For the velocity field of the polymer droplets, due to the high dynamic viscosity existing within polymer droplets, the flow re-directed phenomenon of the droplets is suppressed, which makes this outward motion insufficient to separate the coalesced droplets. As the surface tension decreases, as seen in Figure 9b–d, the velocity inside the droplets increases, the stagnant flow region at the center of droplets decreases (from t* = 1.13 to 2.26), and the negative pressure region inside the droplets increases (from t* = 2.26 to 7.92). From t* = 7.92 to 16.40, the pressure inside of the droplets was changed from a negative pressure to a positive pressure, and the positive pressure decreases as the surface tension decreases (t* = 16.40). The research of Jiang et al. [9] showed that during the subsequent recovery process for the deformed droplets to resume their spherical shape, the pressure inside of the droplets will be increased through the action of the surface tension.

**Figure 10:**
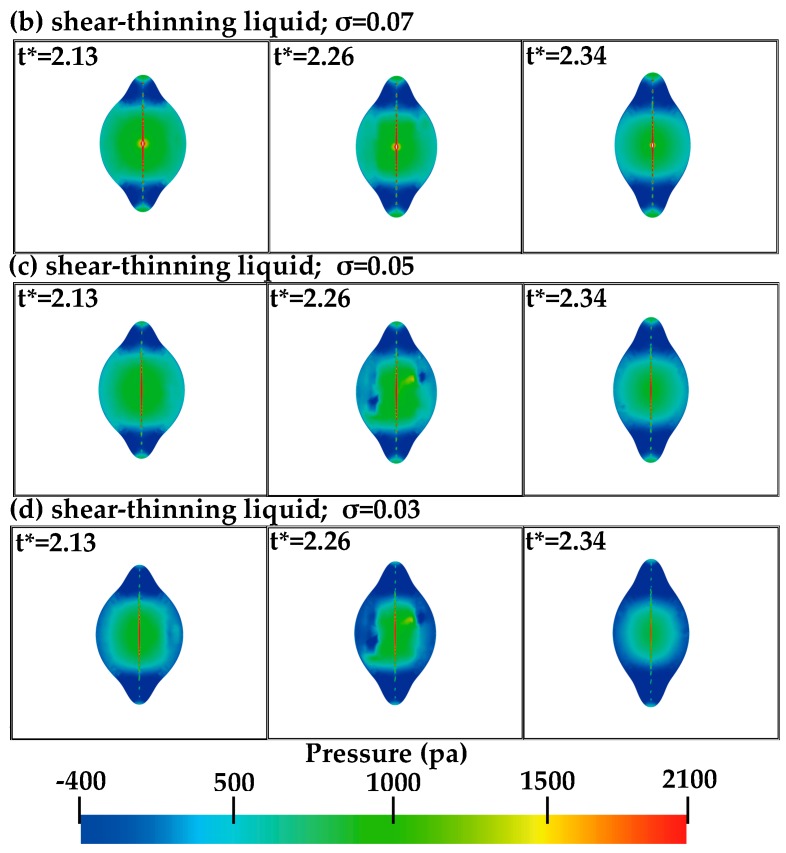
The pressure field for the *Y*–*Z* plane at *X* = 0.00168 of the polymer droplets at t* = 2.13, 2.26, and 2.34, respectively.

In Figure 9b–d, under different surface tensions, some disturbance can be observed at t* = 2.26. The pressure fields of the polymer droplets at collision time near t* = 2.26 are further given in Figure 10.

In Figure 10, at t* = 2.13, the pressure changes are smooth, and no disturbance can be observed, which implies the mechanism of the rupture of the inter-droplet gas film may be related to plane-rupture. This is also observed by Li et al. [20] for the Newtonian liquid. At the low surface tension in Figure 10c,d, the pressure distributes asymmetrically near the center of the droplets, and the pressure changes are not smooth, as shown at t* = 2.26. While at t* = 2.34, the pressure becomes smooth again. This phenomenon of the pressure changes can be explained in that as the surface tension decreases, the Weber number increases, and the interface front can still get the kinetic energy from other parts of the droplets, moving forward slowly and asynchronously [20].

### 4.4. Energy Evolution

Based on the researches of Sarroka et al. [21] and Masato et al. [22], the energy evolution of the droplets in the collision process is further discussed.

The initial kinetic and surface energy of the droplets is computed as

(16) $$E_{K0}=\rho_{l}\frac{\pi }{24}D^{3}V_{r}^{2}$$

(17) $$E_{S0}=2\pi D^{2}\sigma$$

The kinetic and surface energy at each time step during the collision process is computed as

(18) $$E_{K}=\frac{1}{2}\overset{N_{cells}}{\underset{ijk=1}{\sum }}\left(f_{ijk}\rho v_{cell}\right)\left(u_{ijk}^{2}+v_{ijk}^{2}+w_{ijk}^{2}\right)$$

(19) $$E_{S}=\sigma \overset{N_{polygons}}{\underset{p=1}{\sum }}SA_{p}$$

where *SA_p_* is the polygon area and *ν_cell_* is the volume of the computational cell. *f_ijk_* is the volume fraction of the liquid phase, *u_ijk_*, *ν_ijk_*, and *w_ijk_* is the velocity component and *N_cell_* is the number of the computational cell.

Based on the conservation of energy, the dimensionless dissipation energy (*Φ*) of the droplets is defined as

(20) $$\Phi =E_{K}+E_{S}-E_{K0}-E_{S0}$$

**Figure 11:**
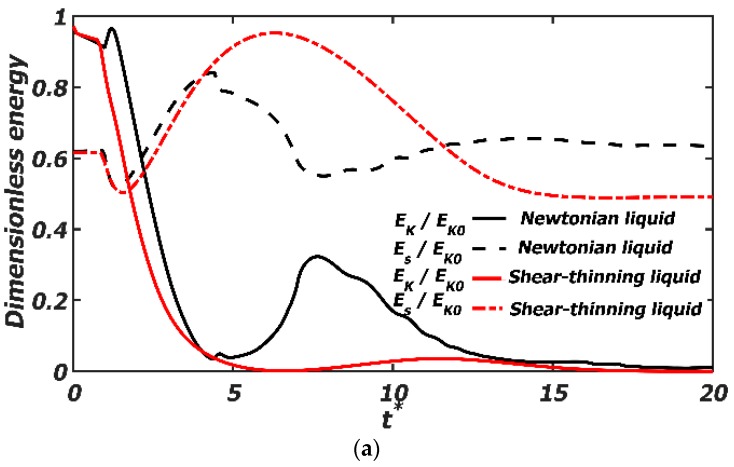

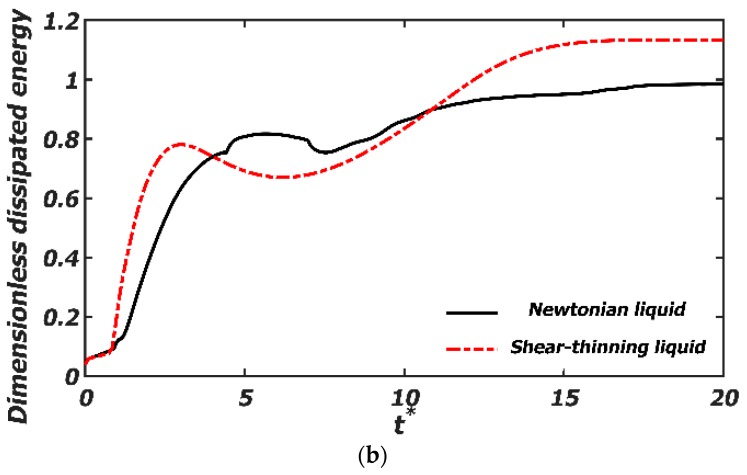
Dimensionless energy evolution the droplets. The Newtonian droplets at *We* = 61.4, *Oh* = 0.0262; the shear-thinning droplets at *We* = 61.4, *Oh*_eff_ = 0.0262. (**a**) Dimensionless time evolution of the dimensionless kinetic energy (*E*_K_/*E*_K0_) and surface energy (*E*_S_/*E*_K0_); (**b**) dimensionless time evolution of the dimensionless dissipated energy (*Φ*/*E*_K0_).

**Figure 12:**
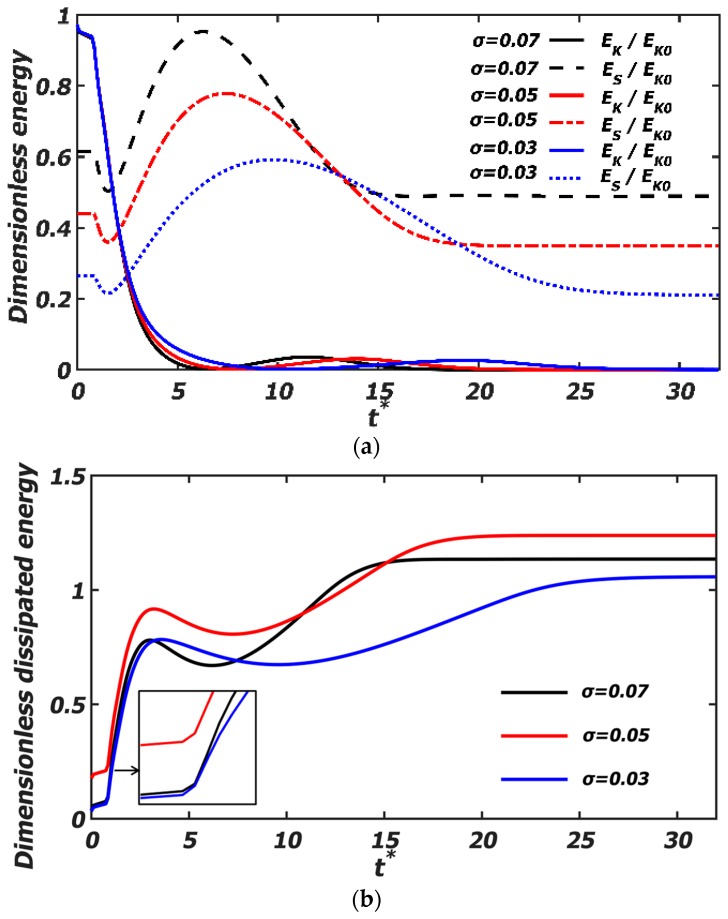
Dimensionless energy evolution of the polymer droplets under different surface tensions (*σ* = 0.07, *We* = 61.4, *Oh*_eff_ = 0.0262; *σ* = 0.05, *We* = 86.1, *Oh*_eff_ = 0.0309; *σ* = 0.03, *We* = 143.5, *Oh*_eff_ = 0.0399). (**a**) Dimensionless time evolution of the dimensionless kinetic (*E*_K_/*E*_K0_) and surface energy (*E*_S_/*E*_K0_); (**b**) dimensionless time evolution of the dimensionless dissipated energy (*Φ*/*E*_K0_).

The kinetic energy (*E*_K_), the surface energy (*E*_S_), and the dissipation energy (*Φ*) are dimensionalized by the initial kinetic energy (*E*_K0_). The dimensionless energy evolution of binary droplets with the dimensionless time is given in Figure 11 and Figure 12.

In Figure 11 and Figure 12, a similar energy evolution process can be observed. At the contact time of the binary droplets, the surface area and the surface energy decreases slightly. As the kinetic energy is transformed into the surface energy, at the instant of the maximum radial deformation of the droplets, the surface energy increases to the maximum, and the dimensionless kinetic energy decreases to the minimum. After that, under the action of the surface tension, the droplets recover the spherical shape, and the surface energy is transformed into the kinetic and dissipated energy. As the droplets continue to extend axially, the kinetic energy approaches zero and the surface energy tends to a steady value. During the collision process, the dimensionless dissipated energy is not monotonously increasing but can show a local extremum. The dissipated energy rises fast at the initial contact stage, and then decreases to the local minimum in the process of the radial contraction, rising again with the axial extension of the droplets.

In Figure 11a, at the initial collision stage, the kinetic and surface energy of the non-Newtonian droplets decreases faster than that of the Newtonian droplets. At the instant of the maximum deformation, for the non-Newtonian liquid, the surface energy reaches 90% of the initial energy, and the kinetic energy approaches zero. While for the Newtonian liquid, the surface energy reaches 80% of the initial energy, and the kinetic energy approaches 10% of the initial energy. Then in the process of the radial contraction, the kinetic energy and the surface energy of the Newtonian droplets take on opposite variation trends, and the surface energy tends to approach the initial kinetic energy. For the non-Newtonian liquid, the surface energy eventually tends to 50% of the initial energy, and the kinetic energy approaches zero value. This obvious change in the surface energy indicates that compared with the Newtonian liquid, there is a larger deformation for binary non-Newtonian droplets collision. In Figure 11b, at the initial stage, the dissipated energy of the non-Newtonian droplets increases faster than that of the Newtonian liquid. This can be used to explain that the polymer droplets coalesce more slowly than the Newtonian droplets in Figure 6. At the final collision stage, the dissipated energy of the Newtonian droplets is almost equal to the initial kinetic energy, while the dissipated energy of the non-Newtonian droplets tends to be stabilized at a higher value. This can indicate that in Figure 5b and Figure 7, the separation of the polymer droplets is almost impossible. This experimental research of Finotello et al. [24] also showed that in the collision process, the kinetic energy was dissipated due to viscous flow, and thus the Weber number for the reflexive separation increased with the Ohnesorge number (*Oh*).

Changing the surface tension of the polymer droplets in Figure 12a shows that the variation of the surface energy is more remarkable, while the variation of the kinetic energy is slight. At the initial radial extension stage, the larger the surface tension is, the larger the surface energy is. At the later collision stage of the droplets, the larger the surface tension is, the faster the surface energy decreases and stabilizes. This can correspond to the process of the radial contraction and the formation of the stable droplets in Figure 7. Moreover, from the kinetic energy evolution curve, it can be observed the kinetic energy at the final time was always less than 10% of the initial kinetic energy. The research of Finotello et al. [23] showed that a collision can be considered completed when the residual kinetic energy was negligible. In Figure 12b, at the initial stage, as the surface tension decreases, the dissipated energy increases, so the coalescence time of the droplets in Figure 8 is delayed. While at a lower surface tension (*σ* = 0.03), the dissipated energy decreases, which can be explained that as the surface tension decreases, the decreased surface energy is far larger than the increased kinetic energy.

## 5. Conclusions

Based on the coupled level-set and volume of fluid (CLSVOF) method and adaptive mesh refinement (AMR), the collision process of the polymer droplets was compared with that of the Newtonian droplets. The effects of surface tension on the collision results of the polymer droplets were further studied. Finally, the internal flow fields (velocity and pressure) and the energy evolution of the droplets were discussed in detail. The conclusions are summarized as follows:

(1) For binary polymer droplets collision, permanent coalescence occurs. Compared with the Newtonian liquid, the maximum deformation of the polymer droplets is promoted, and the colliding droplets coalesce more slowly. By decreasing the surface tension, the maximum deformation is promoted, and the coalescence time for the droplets is delayed.
(2) Compared with the Newtonian liquid, at the initial stage, the larger high-pressure and stagnant flow region appears near the center of the polymer droplets. During the collision process, the flow re-direction phenomenon is suppressed, which makes that the separation is hardly possible. By decreasing the surface tension of the polymer droplets, the stagnant flow region at the center of the droplets decreases. At a low surface tension, due to the non-synchronization in the movement of the interface front, the pressure is not smooth and distributes asymmetrically near the center of droplets.
(3) The energy evolution shows that the dissipated energy of the shear-thinning liquid increases faster than that of the Newtonian liquid. By decreasing the surface tension of the polymer droplets, the surface energy decreases, and the dissipated energy increases. At a low surface tension, due to the decreased surface energy being far greater than the increased kinetic energy, the dissipated energy decreases.

In view of the practical application background of polymers in engineering, the collision process of the droplets under more complex conditions, including binary droplet (including oil–water emulsion) collisions under high pressure, binary droplet collisions under different ambient temperatures, and binary collision of the droplets containing additives, deserve further exploration and breakthroughs.

## Figures and Tables

**Table 1:** Parameter settings in the simulation cases.

Case	Liquid	*σ* (kg/s^2^)	*V*_r_ (m/s)	*Oh*_eff_	*We*	*Re*	*Ca*
A	Newtonian liquid	0.0265	2.52	0.0262	61.4	301.60	0.202
B	Shear-thinning liquid	0.07	3.58	0.0262	61.4	491.56	0.1251
C	Shear-thinning liquid	0.05	3.58	0.0309	86.1	491.56	0.1752
D	Shear-thinning liquid	0.03	3.58	0.0399	143.5	491.56	0.2920
